# Thermal Conductivity Calculation in Organic Liquids: Application to Poly-*α*-Olefin

**DOI:** 10.3390/molecules29020291

**Published:** 2024-01-05

**Authors:** Jonathan Severin, Sophie Loehlé, Philippe Jund

**Affiliations:** 1 Institut Charles Gerhardt Montpellier, UMR 5253 CNRS-UM-ENSCM, 1919 Route de Mende, 34293 Montpellier, France; severin.jonathan@gmail.com; 2 Total Research Center, Chemin du Canal BP 22, 69360 Solaize, France; sophie.loehle@total.com

**Keywords:** thermal conductivity, heat transport, NEMD, molecular dynamics, molecular liquid, lubricant

## Abstract

In this work, we aim to understand and predict the thermal properties of automotive lubricants using non-equilibrium molecular dynamics. After a previous study on model materials for the mechanical parts of a car engine, we now focus on the thermal conductivity κ of the poly- α -olefin base oil (PAO4) using the well-known sink and source method to study the response of the system to an imposed heat flux. We present a detailed methodology for the calculation of κ , taking into account specific constraints related to the system under study, such as large steady-state fluctuations and rapidly growing stationarization times. We provide thermal conductivity results using four different force fields, including OPLS-AA, PCFF and COMPASS, in a temperature range of 300 to 500 K, which corresponds to the typical operating range of a car engine. The results are compared to experimental measurements performed on the commercial compound using the laser flash method. Agreement at room temperature is shown to be excellent for our in-house force field.

## 1. Introduction

Thermal transport has been modeled at all scales, from first-principles calculations to finite-element simulations, by countless researchers. This scientific interest is motivated by a broad range of applications, including (but not limited to) nanoelectronics [1], aerospace [2], automotive [3] and building sector [4]. In order to design or improve industrial systems and processes, understanding and predicting the thermal properties of materials often constitute a key advantage, increasing efficiency and reducing development costs. Nonequilibrium molecular dynamics (NEMD) methods have been applied to thermal conductivity calculations for more than three decades [5], and a number of different algorithms have emerged [6].

Two primary approaches can be distinguished: one involves imposing a temperature gradient, while the other involves imposing the heat flux. The latter method offers the advantage of quicker convergence [7]. Currently, two commonly used algorithms within this approach include the particle velocity exchange introduced in Reference [8] and the heat source and heat sink technique outlined in References [9,10,11]. In this context, we employ the source and sink method, initially developed for amorphous materials and simple Lennard–Jones (LJ) liquids, which has since been adapted to simple crystal structures, encompassing various FCC arrangements [12,13,14,15]. It is worth noting that this method has been extended to more intricate structures like tetragonal ZrO_2_ [16] and zeolitic imidazolate frameworks [17] in select cases.

The work reported here is part of a larger effort which aims to predict the material–lubricant interactions involved in the operation of a car engine by numerically studying the thermal transport in the two phases and at the interfaces.

As such, as a first step, we focused on developing a methodology that we applied to iron oxide (hematite) as a model material for the solid parts of the engine [18], since engine parts in contact with the lubricant are mainly composed of stainless steel, which has already been modeled by hematite (0001) in other studies [19,20,21].

**Figure 1:**
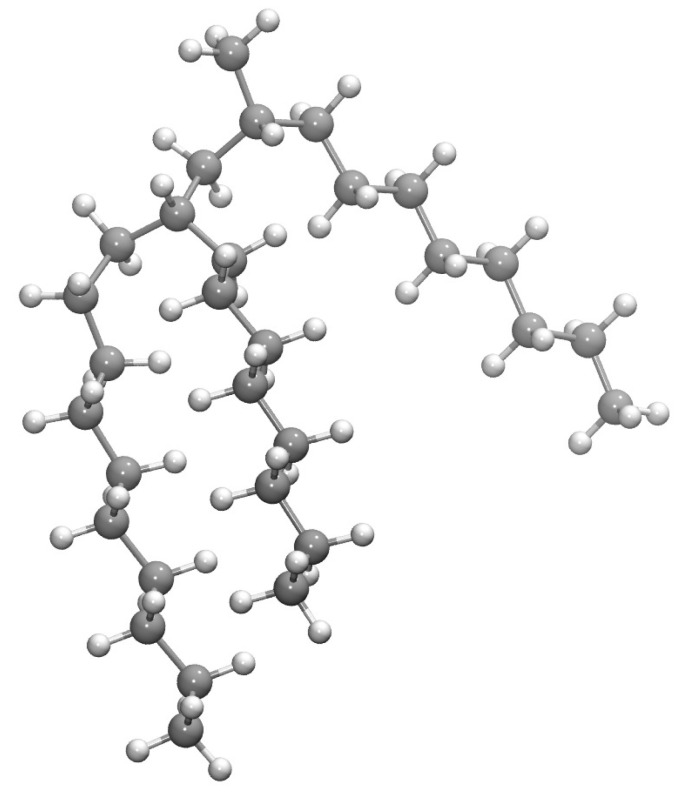
Isolated model molecule of formula C_30_H_62_ representing PAO4 relaxed at room temperature. Carbon atoms are in gray and hydrogen atoms in white.

We now concentrate on the thermal transport properties of the lubricant, focusing specifically on the synthetic poly- α -olefin base oil (PAO4). Commercial lubricants are usually made of a base oil (70–99%) and a set of additives (1–30%) which enhance and complement the properties of the base oil. They can act, for example, as friction modifiers or viscosity improvers [22]. In the automotive industry, the most commonly used synthetic base oil is poly- α -olefin (PAO) which is synthesized from the oligomerization of olefin monomers consisting of linear hydrocarbon chains. The PAO acronym is typically followed by a number describing the kinematic viscosity of the compound: PAO4, PAO6, etc. PAOs can be used for engine oils, grease or other industrial uses. PAO4 has been modeled by C_30_H_62_, as shown in Figure 1, since C_30_H_62_ isomers are the main components of PAO4 [23]. The main objective of the study was to adapt the methodology previously developed for anisotropic crystals to the calculation of the thermal conductivity of PAO4, taking into account the influence of sample size and temperature. We also present a comparison between different force field models for the thermodynamic and thermal transport properties of the compound. The calculated values will be compared to experimental measurements performed on pure C_30_H_62_ PAO4 using the standard laser-flash method at 300 and 450 K.

The paper is structured into three sections. The initial section covers an overview of the NEMD algorithm and methodology, the application of parameterized force fields for lubricant modeling, numerical methods implemented to address sample size effects and the experimental approach utilized for thermal conductivity measurements. Moving to the second section, we delve into the presentation and discussion of our findings concerning the thermal conductivity of PAO4 and its correlation with temperature variations. We also provide a force field comparison and we discuss several elements of the methodology, such as the attenuation of the large steady-state fluctuations and the influence of the box length on the achievement of that steady state. Finally, we draw the major conclusions in the last section.

## 2. Results and Discussion

### 2.1. Density Comparison between Force Fields

Molecular dynamics relaxation runs were performed using the four different force fields described in Section 3.2 to assess the equilibrium density and compare its value to experimental measurements. The simulations were conducted in the NPT ensemble for 1 ns on a small box of approximately 7000 atoms. The results are presented in Table 1. They show that the in-house potential is suitable at room temperature but significantly differs from density measurements at high temperature. When considering the more complex force fields, as expected (see Section 3.2), PCFF underestimates the equilibrium density while COMPASS and OPLS-AA are closer to the mark. As the density results are different from one force field to the other, we found it interesting to have a look at the computational performances of each of them. From the same initial state and on the same machine, the four potentials performed as indicated in Table 2 where a higher number of steps per second indicates a better performance. As expected, the most simple model is faster by more than 40%. OPLS comes second, with a small advantage on PCFF and COMPASS, which are probably slowed down by their cross-term potentials. In the next parts of this paper, we will present thermal conductivity calculations performed mainly with the in-house potential and we will provide a comparison with the other three as well, since those are frequently used in the literature.

### 2.2. Size-Dependent Simulations

**Figure 2:**
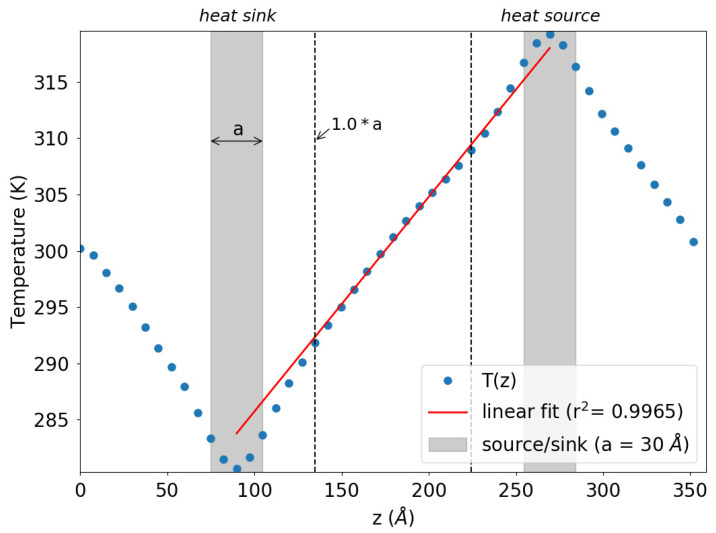
Representative simulation outcome depicting the time-averaged temperature profile (illustrated by blue dots) relative to the position along the z-axis, aligned with the heat flux direction. The gray-shaded sections denote the regions corresponding to the heat sink and source ( $\Delta \epsilon =+/-5\times 10^{-5}$ eV). The dotted vertical lines delineate the boundaries of the linear domain, within which the curve’s slope is computed using a fitting procedure.

As detailed in Section 3, computing κ involves two sequential steps. Initially, a series of size-dependent simulations is conducted, followed by a subsequent size-independent extrapolation. Illustrated in Figure 2 is a representative outcome from an individual simulation concerning a specific box size *L*. The visual showcases the time-averaged temperature profile alongside the placement and width of the slabs designated as the heat source and heat sink. Notably, within the central section of the simulation box, a linear temperature profile is evident, facilitating the calculation of the thermal gradient through fitting. Regions exhibiting non-linear temperature profiles are observed in proximity to the heat source and sink locations.

**Figure 3:**
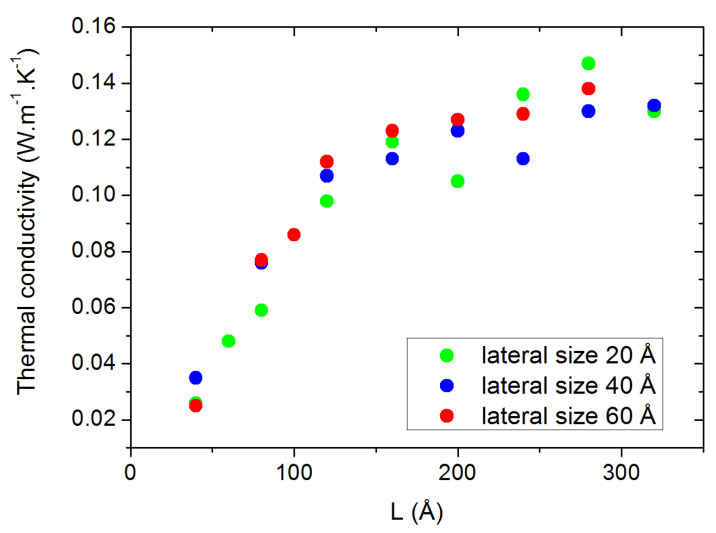
Thermal conductivity at 300 K for various lateral box sizes, presented as a function of the box size in the heat flux direction.

**Figure 4:**
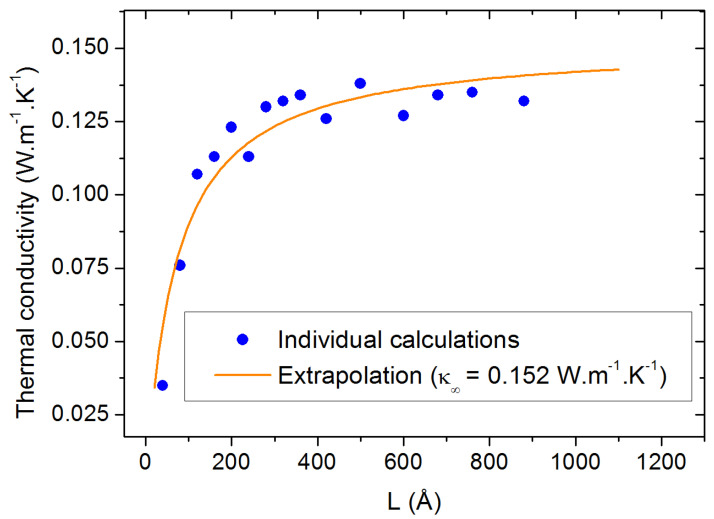
Thermal conductivity calculation at 300 K as a function of the box size parallel to the heat flux. The orange line is the result of the extrapolation procedure.

Concerning the size effects, we first investigated the influence of the lateral sizes (orthogonal to the heat flux) by calculating the thermal conductivity as a function of the longitudinal size *L* for three different lateral sizes (20, 40 and 60 Å). From the results shown in Figure 3, no significant differences can be traced back to the size of the lateral dimensions, except a decrease in the statistical dispersion for the largest sizes. Therefore, we chose to fix the lateral size to 40 Å for the rest of the study to compromise between statistical accuracy and computational requirements. As for the evolution of κ as a function of *L*, the size parallel to the heat flux (horizontal axis in Figure 3), it requires the application of the extrapolation method discussed in Section 3.3. The result of this extrapolation is shown in Figure 4 along with all the calculated values of κ for individual values of *L*. The corresponding value of $\kappa_{\infty }$ is calculated to be 0.152 W.m^−1^.K^−1^ for pure PAO4 at 300 K. This value compares extremely well with the values of 0.149 W.m^−1^.K^−1^ which were experimentally measured at room temperature with the standard laser flash method [24,25].

### 2.3. Temperature Dependence and Force Field Comparison

**Figure 5:**
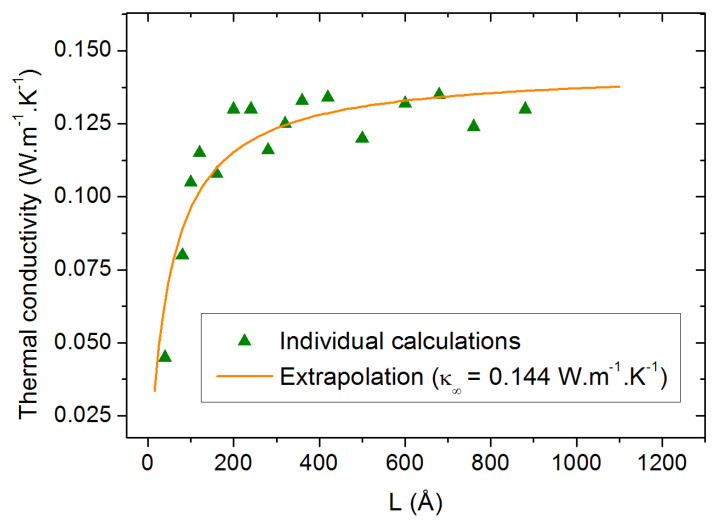
Thermal conductivity calculation at 500 K as a function of the box size parallel to the heat flux. The orange line is the result of the extrapolation procedure.

In order to determine the temperature dependence of κ , we performed a similar study at 500 K. The results are shown in Figure 5. The corresponding value of $\kappa_{\infty }$ is calculated to be 0.144 W.m^−1^.K^−1^. This is significantly larger than the value of 0.124 W.m^−1^.K^−1^ measured experimentally at 450 K but it is coherent with the large equilibrium density obtained at 500 K (Section 2.1).

**Figure 6:**
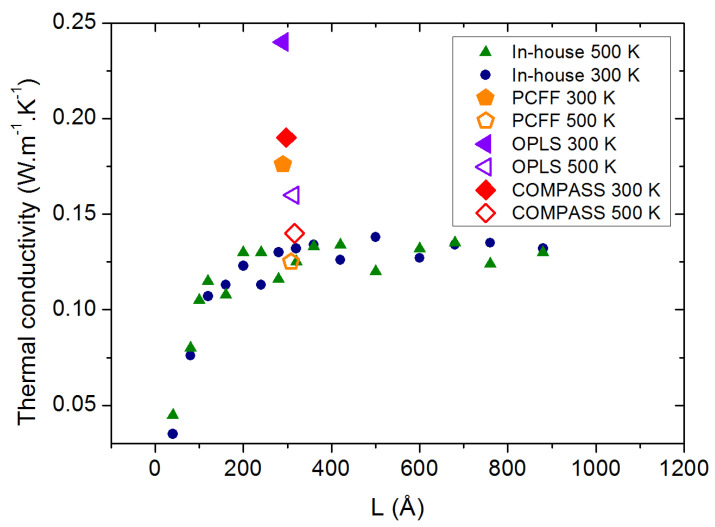
PAO4 thermal conductivity calculated with 4 different force fields at 300 and 500 K.

The discrepancy between our calculations and the measurements at high temperature led us to compare the thermal conductivity values obtained with the in-house force field with new calculations using OPLS, PCFF and COMPASS. As can be observed in Figure 6, these potentials exhibit the expected decrease in the thermal conductivity as the temperature is raised from 300 to 500 K, but all three of them present a strong overestimation of κ at both temperatures, which is a strong drawback as well.

### 2.4. Remarks on Methodology

**Figure 7:**
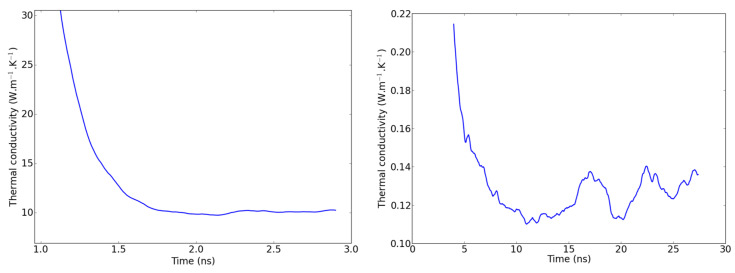
Thermal conductivity fluctuations during the production run in an NEMD simulation of Fe_2_O_3_ (**left**) and PAO4 (**right**).

**Figure 8:**
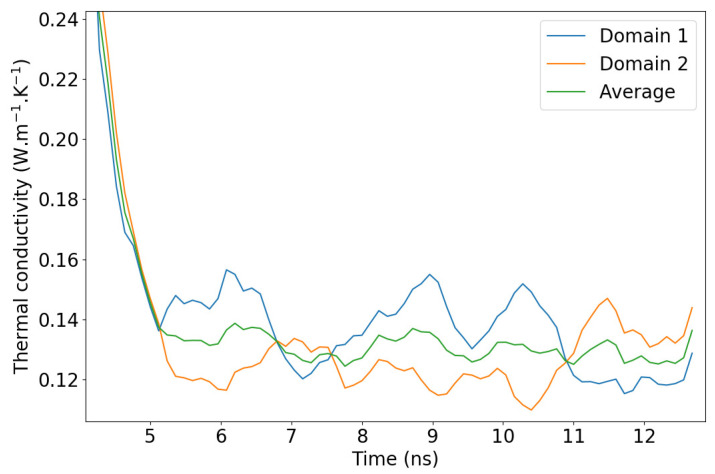
PAO4 thermal conductivity fluctuations during stationarization and production. The red line is the average that was calculated using both domains of the simulation box simultaneously.

When applying the NEMD scheme to a weakly conductive liquid, one should expect the methodology to be impaired by fluctuations in the temperature profile, which cause the thermal conductivity to fluctuate slightly even when the steady state is supposed to be reached. In Figure 7, a comparison is proposed between the evolution of the NEMD-calculated thermal conductivity with time for a single-crystal hematite on the one hand and PAO4 on the other hand. It is clearly more difficult to assess a precise value of κ from the second graph of Figure 7 where large fluctuations have to be attenuated with long time averages. Investigating these fluctuations, we observed that they are not strictly caused by the higher particle mobility in liquids compared to solids. In fact, keeping in mind that the simulation box is divided into two domains with two temperature gradients, we determined that the largest fluctuations compensate each other when considering the two domains at the same time. For the sake of clarity, we should hereafter refer to the central part of the simulation box (between the dotted vertical lines of Figure 2 as *domain 1* and to the analogous part that is spread across the periodic boundaries as *domain 2*. In other words, when the temperature gradient rises in *domain 1*, it simultaneously goes down in *domain 2*, as shown in Figure 8. One possible explanation for this is an unequal division of the instantaneous heat flow between the left and right side of the heat source. With this new observation, we recommend that the thermal conductivity be averaged on both domains of the box when working with systems prone to large fluctuations i.e., systems with small thermal conductivities.

**Figure 9:**
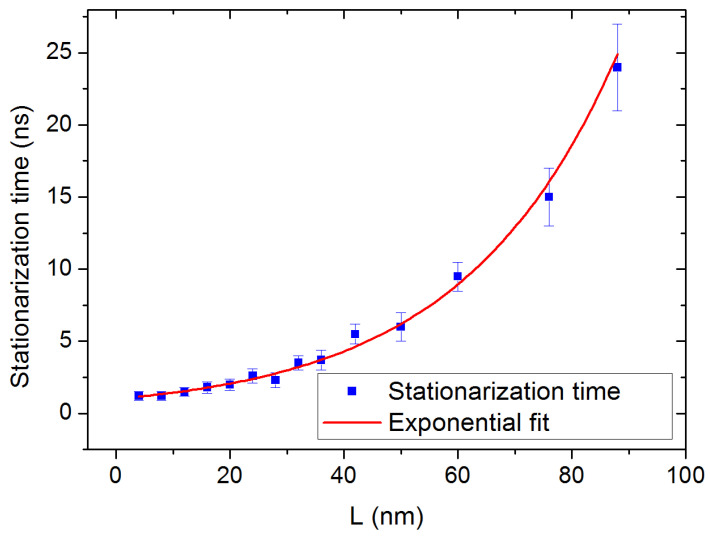
Time necessary to achieve the steady state as a function of the box length *L* (blue squares). To emphasize the exponential growth of the stationarization time, the data points are fitted with a function of the form $y=e^{\frac{x}{l}}$ where the length *l* is equal to 27 nm. This function tends to 1 ns when the size tends to 0, exhibiting a finite minimum relaxation time.

Another observation is that the time necessary to achieve the steady state, after activation of the heat flux, strongly depends on the length *L* of the simulation box. Figure 9 shows the fast increase in the stationarization time as a function of *L*, reaching 25 ns for the largest sizes. This is contrary to the observation made in most of the studies on the subject, in which the stationarization time does not depend significantly on the box size: a value of approximately 1 ns has been reported for silicon [6,26], a stationarization time of 0.8 ns has been observed for iron oxide [18], and Hu et al. report approximately 2.5 ns for water/silica interfaces [27]. Finally, NEMD has been applied to liquid n-alkanes [28] and the stationarization time is presented as related to the hydrocarbon chain length, not to the box size. This exponential growth might constitute a considerable limitation for similar compounds with higher thermal conductivities for which larger box sizes would be required, since a priori the MFPs of the heat-carrying phonons would be larger.

## 3. Methods

### 3.1. Molecular Dynamics and NEMD

Classical molecular dynamics (MD) stands as a widely embraced method offering intricate atomic-level insights into material characteristics and processes. By integrating Newton’s equations of motion at the atomic scale, this method enables the utilization of relatively large simulation boxes, a notable advantage over first-principles calculations. In non-equilibrium molecular dynamics, one or more internal variables are deliberately constrained to maintain the system in a non-equilibrium state.

In our case, we use the standard velocity Verlet algorithm for the time integration with a time step of 0.6 fs, which was optimized after studying energy conservation at small and long time scales. In addition, we apply the method described in [10] for the determination of the thermal conductivity κ . For sake of explanation, let us consider a heat flux applied along the z direction of the simulation box and *L* the size of the box in that direction. We then establish two slabs, each with a width *a*, positioned at 1/4 and 3/4 of *L*, respectively, orthogonal to the z-axis. To maintain continuity, we apply periodic boundary conditions (PBC) in all dimensions. In each time step, a fixed amount of energy Δϵ is introduced to one of these slabs, while an equal amount is extracted from the other. This process involves constant rescaling of atom velocities within the slabs, effectively enforcing a heat flux across the box along the z-axis. Measures are implemented to prevent any center-of-mass drift arising from velocity rescaling. For a particle i within the hot slab $P_{+}$ , the velocity rescaling can be articulated as follows:

(1) $${\bar{v}}_{i}=v_{G}+\alpha \left(v_{i}-v_{G}\right)$$

where ${\bar{v}}_{i}$ is the updated velocity, $v_{G}$ the velocity of the center of mass of the ensemble of particles in $P_{+}$ and

(2) $$\alpha =\sqrt{1+\frac{\Delta \epsilon }{E_{P_{+}}}}.$$

Naturally, when considering the cold plate, denoted as $P_{-}$ , the value of Δϵ should be subtracted. The non-translational kinetic energy $E_{P_{+}}$ is calculated as

(3) $$E_{P_{+}}=\frac{1}{2}\sum_{i}m_{i}v_{i}^{2}-\frac{1}{2}\sum_{i}m_{i}v_{G}^{2}.$$

**Figure 10:**
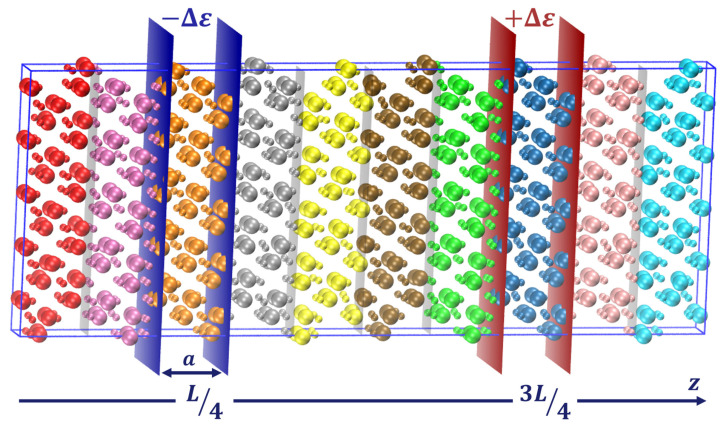
Schematic representation of the NEMD method. The temperature slices are represented (colored regions) along with the heat source and sink (enclosed between the red and blue slabs, respectively).

To monitor the temperature distribution along the z-axis, the box undergoes segmentation into multiple slices where local temperatures are computed, as depicted in Figure 10. Since the number of atoms per slice is directly related to the density, the width of these slices directly impacts the accuracy of the temperature calculations. A larger number of atoms provides better statistics and so allows us to reduce the time over which the values need to be averaged. But to obtain a detailed temperature profile, the number of slices has to be large enough and therefore their width is limited. In order to eliminate this constraint, we follow the approach defined in our previous study: 4 sets of 12 bins of *a* in width are defined with an overlapping distance of a/4 ; more details can be found in [18].

As mentioned earlier, the PAO4 molecule is made of hydrocarbon chains, and obtaining a proper initial state for a liquid of such nature is not trivial. First of all, a small cubic box of approximately 1000 atoms is built at the desired density using the Amorphous Cell module of the Materials Studio computational package [29]. A Monte Carlo-like approach allows us to randomly build one molecule at a time, checking the energy of the new configuration as well as close contacts with the atoms already present. During this process, the energy is evaluated using the COMPASS force field and energy minimization is performed to obtain a low-energy initial state for the future simulations. The result is a dense state with chain entanglements that can then be replicated to provide different system sizes. Throughout the simulations, the system initiates with a brief run in the NVT ensemble lasting 500 ps, followed by an extended NPT relaxation lasting 2 ns at the desired temperature. This stage ensures the convergence of both potential energy and density within the system. Once the system reaches a stable relaxed state, the heat flux is activated. Our investigations confirm that minor variations in the energy exchanged between the slabs do not notably impact the value of κ . However, substantial values of Δϵ can result in considerable temperature differences between the heat source and sink, thus warranting caution, especially if phase transitions are anticipated around these temperatures. As a rough guideline, about 0.2% of $k_{B}T$ appears to be a reasonable parameter for a simulation box housing ≈ 70,000 atoms, utilizing the described heat sink and source configuration. Upon activating the heat flux, allowing sufficient time for the system dynamics to attain a stable state becomes imperative. Typically, achieving this steady state takes around 1 ns, as observed in studies on systems like Stillinger–Weber silicon [6,26]. However, as discussed in Section 2.4, determining the time required for the PAO4 molecule under investigation to reach a steady state is not as straightforward. The subsequent phase in the simulation involves the production run: while the heat flux remains active, we initiate the collection and averaging of temperature values across each slice. Depending on the system’s scale, we observe a variation in the time required for averaging to mitigate statistical noise and yield a smooth temperature profile suitable for accurate linear fitting, ranging between 5 and 30 ns. Both the stationarization and production runs operate within the microcanonical ensemble (NVE). Subsequently, the temperature gradient is computed from the time-averaged profile, and Fourier’s law (4) is employed to derive κ . Wirnsberger et al. [30] introduced an adapted heat exchange algorithm to rectify long-term simulations’ total energy drift. However, in our study, we notice a moderate disparity between initial and final total energy values (approximately $10^{-3}$ % over a 10 ns run), which does not necessitate the use of an enhanced algorithm.

(4) $$\vec{J}=-\kappa \vec{\nabla }T$$

The utilization of a method akin to the one outlined in this study exerts a notable impact on computational performance. The continuous temperature rescaling within the heat source and sink, coupled with the local temperature computations across each slice, significantly contribute to computational time consumption. Additionally, considering the imperatives of achieving stationarity and averaging, the computational (and real-time) expenses become a primary concern. Notably, certain simulations conducted as part of this research demanded more than 150,000 CPU hours each. These molecular dynamics simulations were executed using a modified iteration of the LAMMPS package [31].

### 3.2. Interaction Potential

To perform molecular dynamics on a system made of PAO molecules, one needs to apply a molecular force field comprising bonded and non-bonded interactions. We made use of three of the most popular force fields typically applied to this kind of system (OPLS-AA, PCFF and COMPASS), which we compared to a more simple, in-house, potential. The all-atom optimized potential for liquid simulations (OPLS-AA) [32,33,34] was developed based partly on the AMBER all-atom force field [35] and on ab initio and Monte Carlo calculations for a large number of organic liquids and proteins. Intramolecular interactions are modeled with bond stretching and angle bending by harmonic potentials and dihedral angles by a cosine series. Atoms separated by three bonds also interact with a 12-6 Lennard–Jones potential scaled by 0.5 and an electrostatic interaction scaled by 0.5 [36]. PCFF and COMPASS are more complex force fields: in addition to the interactions described above, anharmonic and cross-terms potentials are added. They both share the same functional form but use different sets of parameters obtained with different fitting methods. The polymer consistent force field (PCFF) was developed based on CFF91 and CFF93, initially for a large group of organic and inorganic polymers [37]. The parameters were derived from ab initio calculations and experimental data using 0 K energy minimization techniques, leading to a certain degree of error for thermodynamic properties such as equilibrium density when performing MD simulations [38]. The condensed-phase optimized molecular potential for atomistic simulation studies (COMPASS) inherited some of the parameters from PCFF, but its development included a refinement step where non-bonded interaction parameters were adjusted using finite-temperature MD simulations. This enhanced fitting approach, along with the transfer of many parameters from one functional group to another, improved the accuracy of the thermodynamic properties and provided better versatility regarding the applications of the force field [38].

Finally, an in-house potential (*U*) was developed starting from the consistent valence forcefield (CVFF) [39]. The force field was fitted to amides, carboxylic acids and other small organic molecules in crystal and gas phase structures. It was initially intended for the study of structural binding energies, vibrational frequencies and conformational energies. A Morse potential is used for the bond-stretching term and a harmonic potential for the bond angles. Non-bonded interactions are modeled with Coulomb and Lennard–Jones 12-6 (LJ) potentials and the usual 0.5 scaling is applied. The carbon atoms are divided into three types according to their presence in a CH, CH_2_ or CH_3_ group. Compared to the original CVFF parameters, only the parameters of the bond-stretching term and of the non-bonded interactions have been modified to recover the value of the experimental thermal conductivity of PAO4 at 300 K. To avoid a modification of the density, we principally decreased the energy values of the potential well-depths by ≈10% . The new parameters of the two potential terms are specified in Table 3 and Table 4. Finally for the dihedral torsions, the OPLS model and parameters are employed [32] and, as a first simplification, the hydrogen atoms are not taken into account for the LJ interactions and no cross-terms are included in the model.

(5) $$\begin{array}{c} \begin{array}{c} U\left(r,\theta ,\phi \right)=D{\left[1-e^{-a(r-r_{0})}\right]}^{2}+K\left(\theta -\theta_{0}\right)+\frac{1}{2}K_{1}\left[1+cos\left(\phi \right)\right]+\frac{1}{2}K_{2}\left[1-cos\left(2\phi \right)\right]\\ +\frac{1}{2}K_{3}\left[1+cos\left(3\phi \right)\right]+\frac{1}{2}K_{4}\left[1-cos\left(4\phi \right)\right]+4\epsilon \left[{\left(\frac{\sigma }{r}\right)}^{12}-{\left(\frac{\sigma }{r}\right)}^{6}\right]+\frac{z_{i}z_{j}e^{2}}{r} \end{array} \end{array}$$

### 3.3. Finite Size Effects

Heat flow in PAO4 is primarily described by phonon transport. When conducting NEMD within a finite simulation box, the phonon mean free path (MFP) becomes a critical factor. The MFP denotes the average distance a phonon can travel before encountering scattering. Different MFPs among phonons contribute variably to thermal transport, with certain materials exhibiting MFPs extending beyond several hundred nanometers [40,41]. In the context of the NEMD method outlined in earlier sections, defining two slabs at one-quarter and three-quarters of the box length to function as heat exchangers poses a challenge. It necessitates a simulation box twice as long as the longest contributing MFP. Managing scales of this magnitude in molecular dynamics simulations demands substantial computational resources and entails excessively prolonged execution times. Moreover, at the beginning of a study, one does not necessarily know the minimum size at which finite size effects cease to influence the calculated thermal conductivity κ of a given compound. Therefore, those effects have to be characterized before a reliable value of κ can be assessed.

Our investigation focused on assessing the impact of finite box dimensions perpendicular to the heat flux direction, utilizing the approach introduced by Schelling et al. to address finite size effects in the parallel direction of the heat flux [6]. To address the latter, we conducted multiple simulations across various box sizes *L*, employing a linear extrapolation technique to combine their outcomes. As the simulation box size increased, accounting for more phonon mean free paths amplified the calculated thermal conductivity. A linear relationship between 1/κ and 1/L is expected within a first-order approximation [40]. Consequently, we extrapolated the curve to $1/L_{\infty }=0$ by plotting 1/κ against 1/L using a linear function to evaluate $\kappa_{\infty }$ . Achieving a precise curve fit necessitates conducting numerous individual simulations. Nonetheless, the computational demand remains considerably lower compared to simulations, with *L* surpassing the longest phonon mean free path. This method also presents an advantage by deriving a thermal conductivity value based on a substantial number of diverse simulations with varied initial states, thereby reducing statistical errors attributed to individual simulations. However, Sellan et al. [40] noted limitations associated with this extrapolation method, particularly concerning its first-order approximation. For instance, while favorable results were reported for Lennard–Jones argon, an underestimation was observed for Stillinger–Weber silicon. In the latter case, the calculated values and the extrapolated curve would diverge for the largest sizes. Hu et al. [42] proposed a correlation between the divergence and the aspect ratio of the simulation box (length/width) to address this divergence. In their exploration involving Lennard–Jones solid argon, Lennard–Jones WSe_2_, and graphite, they concluded that for elastically isotropic materials like LJ argon, divergence is observed at exceptionally high aspect ratios (200 to 300). Yet, they noted that practical implications of such high limits remain inconsequential, since a converged κ value can be computed well below these limits. Their second observation highlighted clear divergent behavior at low aspect ratios (approximately∼30) for elastically anisotropic materials like LJ WSe_2_ and even earlier for graphite. In conclusion, these limits to the NEMD methods mainly apply to anisotropic materials and should not be an issue when working on liquids, as is the case here.

## 4. Conclusions

We have calculated the thermal conductivity of the PAO4 base oil using four different force fields at 300 and 500 K. The room temperature results using the in-house potential are in excellent agreement with the experimental measurements performed on pure PAO4. However, at 500 K, this force field significantly overestimates κ . This can readily be related to the overestimated equilibrium density observed at that temperature. On the other hand, the alternative force fields (OPLS-AA, PCFF and COMPASS) all present a clear overestimation of the thermal conductivity at low and high temperature. Aside from these results, a detailed methodology has been presented with the addition of two specific observations. The first one is related to the large fluctuations during the thermalization process, which complicate the determination of κ and can be significantly attenuated by averaging over the two symmetrical domains of the simulation box in which an instantaneous compensation phenomenon is observed. The second observation points out a possible limitation of the approach due to a fast increase in the required stationarization time when large box sizes are necessary.

## Figures and Tables

**Table 1:** Calculated and experimental density of PAO4 (kg.m^−3^).

Temp.	OPLS	PCFF	COMPASS	In-House FF	Exp. ^1^
300 K	788	742	789	865	806
500 K	615	595	640	840	679

^1^ values provided by Total M.S.

**Table 2:** Number of time steps per second for each force field.

OPLS	PCFF	COMPASS	In-House FF
61	46	48	76

**Table 3:** In-house force field: Morse parameters.

Atom	D (eV)	a (1/Å)
C1-C2	3.81598	1.915
C1-C3	3.81598	1.915
C1-H	4.70927	1.771
C2-C2	3.81598	1.915
C2-C3	3.81598	1.915
C2-H	4.70927	1.771
C3-H	4.70927	1.771

**Table 4:** In-house force field: LJ parameters.

Atom	z	ϵ (eV)	σ (Å)
C1	−0.053	8.61719 × 10^−4^	4.65000
C2	−0.106	3.96391× 10^−3^	3.95000
C3	−0.159	8.44484 × 10^−2^	2.55485
H	0.053	-	-

## Data Availability

The data presented in this study are available in article.
